# Developing a Thin Film Composite Membrane with Hydrophilic Sulfonated Substrate on Nonwoven Backing Fabric Support for Forward Osmosis

**DOI:** 10.3390/membranes11110813

**Published:** 2021-10-25

**Authors:** Soleyman Sahebi, Mohammad Kahrizi, Nasim Fadaie, Soheil Hadadpour, Bahman Ramavandi, Ralph Rolly Gonzales

**Affiliations:** 1 Research and Technology Centre of Membrane Processes (RTCMP), School of Chemical, Petroleum and Gas Engineering, Iran University of Science and Technology (IUST), Tehran 16846-13114, Iran; m_kahrizi@alumni.iust.ac.ir (M.K.); nasim.fadaiee@gmail.com (N.F.); soheils11@yahoo.com (S.H.); 2 Environmental Health Engineering Department, Faculty of Health and Nutrition, Bushehr University of Medical Sciences, Bushehr 75187-59577, Iran; ramavandi_b@yahoo.com; 3 Research Center for Membrane and Film Technology, Kobe University, Kobe 657-8501, Japan

**Keywords:** forward osmosis, hydrophilic substrate, sulfonated polyethersulfone, thin-film composite, nonwoven-embedded substrate

## Abstract

This study describes the fabrication of sulfonated polyethersulfone (SPES) as a super-hydrophilic substrate for developing a composite forward osmosis (FO) membrane on a nonwoven backing fabric support. SPES was prepared through an indirect sulfonation procedure and then blended with PES at a certain ratio. Applying SPES as the substrate affected membrane properties, such as porosity, total thickness, morphology, and hydrophilicity. The PES-based FO membrane with a finger-like structure had lower performance in comparison with the SPES based FO membrane having a sponge-like structure. The finger-like morphology changed to a sponge-like morphology with the increase in the SPES concentration. The FO membrane based on a more hydrophilic substrate via sulfonation had a sponge morphology and showed better water flux results. Water flux of 26.1 L m^−2^ h^−1^ and specific reverse solute flux of 0.66 g L^−1^ were attained at a SPES blend ratio of 50 wt % when 3 M NaCl was used as the draw solution and DI water as feed solution under the FO mode. This work offers significant insights into understanding the factors affecting FO membrane performance, such as porosity and functionality.

## 1. Introduction

Forward osmosis (FO), as an evolving desalination technology, has received significant research interest in recent years. In this process, no hydraulic pressure is required for filtration to occur; thus, the amount of energy required is much lower in comparison with other pressure-driven desalination technology, such as nanofiltration (NF) and reverse osmosis (RO) [1,2]. Osmosis occurs when a solute with high solubility generates a concentration gradient within a solution. The osmotic pressure gradient functions as the driving force to induce water flow from the low concentrated side to the high concentrated side. To utilize this pressure gradient as the driving force, a suitable semipermeable membrane is required to separate the feed solution (FS) and draw solution (DS) [3]. The water flow through the membrane from the FS to the DS side is spontaneous as a natural consequence of osmotic pressure gradient and will continue until both FS and DS sides reach an osmotic equilibrium [4]. Compared to the pressure-driven membrane processes (RO and NF), the FO process has several advantages, such as lower energy requirements, especially when DS recovery is not required; lower membrane fouling propensity and easier fouling management; competitive contaminant rejection; and applicability for treatment of high-salinity and fouling-prone feed streams, which cannot be treated by other membrane-based technologies [1,5,6,7].

Despite the advantages of FO, this process suffers from a lack of suitable membranes and DS recovery strategies. A suitable FO membrane should be robust and semipermeable, with high water flux, high selectivity against a number of solutes, and chemical and thermal stability [8,9]. Initially, commercial RO membranes were utilized for the FO process until the manufacture of the first industrial FO membrane customized for this process by Hydration Technologies Inc. (HTI, Albany, NY, USA) [10]. The HTI FO membrane was developed based on cellulose triacetate (CTA) polymer with nonwoven fabric support [11]. Currently, the major types of FO membranes include asymmetric membranes, thin-film composite (TFC) membranes, thin film nanocomposite (TFN) membranes, and chemically modified membranes [11,12,13].

Among the different membrane types, TFC membranes are the most conventionally used membranes for the FO process. TFC membranes are mainly made of polyamide (PA) as a rejection layer on porous sub-layers through interfacial polymerization (IP) in both flat sheet [12,13,14] and hollow fiber configurations [15,16]. The TFC FO membrane is typically prepared by casting a polymeric membrane substrate on a nonwomen fabric support backing and the subsequent IP reaction of 1,3-phenylenediamine (MPD) and 1,3,5-benzenetricarbonyl trichloride (TMC) [15,17,18]. Some TFC GO membranes do not employ backing fabric supports; however, without fabric supports, TFC membranes might be compacted under the influence of hydraulic pressure such as pressure-assisted osmosis (PAO) and pressure-retarded osmosis (PRO) [8,19].

Generally, water permeability and selectivity can be enhanced from a porous sub-layer and a thin rejection layer modification. Thus, FO membranes can be improved through surface modification of the rejection layer and porous sub-layer or by embedding support with a suitable backing fabric that does not hinder water flux. However, modifying the porous sub-layer appears to be easier than other modification options. Hydrophilic polymer film can be plasticized when exposed to water, and the rejection layer and backing fabric are more rigid [20]. The main objective of membrane performance enhancement can be achieved through lowering membrane structural parameter (S value). The membrane S value is used to determine the internal concentration polarization (ICP) degree in the porous support structure of FO membranes. The S value is determined by membrane support layer thickness, tortuosity, and porosity. Consequently, as there are several chemical and physical modification strategies and fabrication methods to minimize the structural parameter of FO sub-layer, modifying the TFC FO membrane support has more effectiveness and advantages compared to rejection layer modification [17,21].

Internal concentration polarization (ICP) is a major factor influencing the membrane performance during FO operation [16,21,22]. ICP is related to the substrate structural parameter and chemical properties of the support layer [22]. Moreover, previous studies suggested that a substrate with finger-like morphology is ideal to have high water flux and to decrease the ICP, due to a direct path for water molecules and lower resistance [15]. However, another study showed that having high performance in the FO process is more related to membrane chemical properties (e.g., hydrophilicity) rather than membrane physical properties (e.g., membrane morphology) [23]. It was found that membrane performance was significantly higher in the hydrophilic but sponge-like membrane where both initial polymer dope concentrations used for phase inversion followed by IP were the same. However, due to chemical modification to induce hydrophilicity, membrane tensile strength was substantially decreased. Without backing fabric support, these substrates demonstrated low tensile strength and were not considered fit for commercial production [8,24]. Therefore, there is a need to develop a TFC FO membrane to have both ideal properties of mechanical strength and hydrophilicity for a higher performance.

For developing a high-performance membrane, the substrate structure can be modified. There are several options to increase membrane hydrophilicity, such as nanoparticle incorporation in the membrane during phase inversion and/or IP [25,26,27,28,29] and chemical modification [30]. These can be achieved by (a) adding PEG in the support; (b) incorporating Carbone allotropes and other nanoparticles [17,31]; and (c) fabrication methods with “different solvents” and support post-treatment with “activating solvent” [32]. These methods’ effects on physicochemical properties of the membrane include support pore size, porosity, surface charge, hydrophilicity, and functional groups [33,34]. For example, support pore size in the top skin layer during interfacial polymerization may have both negative and positive impacts on the performance of a TFC FO membrane [35,36]. The membrane support improvement to acquire hydrophilic support with low ICP and low structural parameter can be achieved in several pathways. However, enhancing membrane hydrophilicity through sulfonation seems to be more effective compared to other polymeric support modification methods. The only disadvantage of sulfonation is membrane tensile strength decline, which can be ignored in reinforced support membranes with backing fabric support. Direct and indirect sulfonation has been used in several previous studies as a facile chemical modification method to enhance the membrane hydrophilicity. For instance, sulfonated poly(ether ketone) (SPEK) polymer was blended with polysulfone by Han et al. [37] and resulted in enhanced membrane hydrophilicity and reduced structural parameter.

In this study, PES polymer was blended with sulfonated PES (SPES) to prepare a high-performance FO membrane reinforced on nonwoven polyethylene terephthalate (PET) backing fabric. The highly hydrophilic SPES was synthesized based on previous studies [23,38]. The SPES composition varied from 0 wt % to 50.0 wt %. The effects of SPES content on the hydrophilicity, thickness, and morphology were examined. Finally, the developed TFC membranes with different PES/SPES ratios were assessed in the FO process and compared with a neat TFC membrane.

## 2. Materials and Methods

### 2.1. Chemicals and Materials

Polyethersulfone (PES, Sigma-Aldrich, MO, USA) (Mn: 55,000) was used for the fabrication of membrane substrates. Solvent for casting solution was N-methyl-2-pyrrolidone (NMP, >99.5%, Merck, Dramstadt, Germany). Trimesoyl chloride (TMC) (98% purity, Sigma-Aldrich and *m*-phenylenediamine (MPD) (>99% purity, Sigma-Aldrich) were used for the interfacial polymerization. N-hexane (Sigma–Aldrich 99.0%) was used as the solvent for TMC. Commercial non-woven polyester fabric (PET, Grade 3250 Ahlstrom, Helsinki, Finland) was used as support backing fabric. Sodium chloride (NaCl) was used for preparing the draw solution and feed solution. DI water was used as a feed solution (FS) and NaCl with concentrations of 0.5, 1, 2, and 3 M were used as DS.

### 2.2. Preparing of Sulfonated Polyethersulfone (SPES)

**Figure 1:**
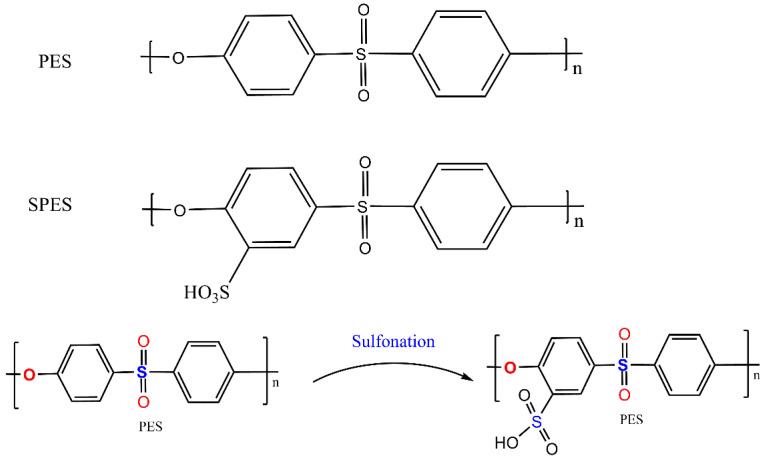
Polyethersulfone (PES) structure and sulfonated polyethersulfone (SPES) chemical structure after sulfonation.

**Figure 2:**
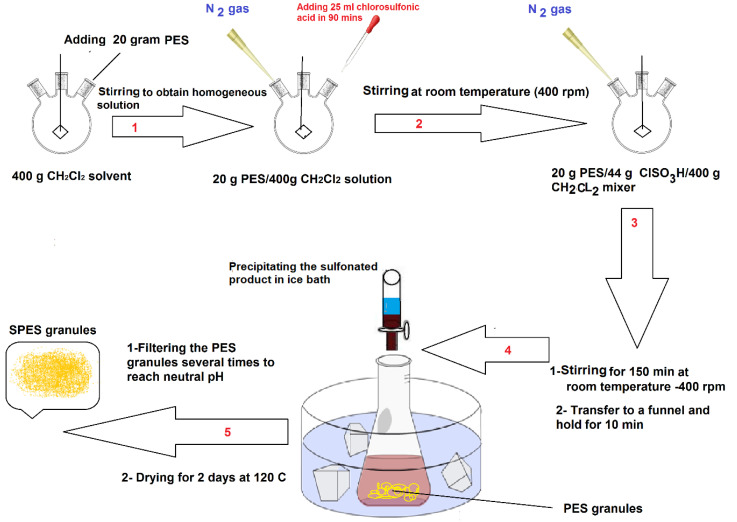
PES sulfonation based on the process by Li et al. [38].

PES chemical structures before and after sulfonation are presented in Figure 1. SPES polymer was prepared based on previous studies [38,39]. Figure 2 shows the step-wise polyethersulfone (SPES) polymer sulfonation procedure.

We mixed 20 g PES with 400 g CH_2_Cl_2_ and stirred continuously to obtain a homogeneous solution. Then, 25 mL chlorosulfonic acid was added in the mixture under N_2_ atmosphere, in the close system. The mixture was placed in stirring conditions at room temperature at 400 rpm for 150 min. The treated mixture was poured into 1000 g of methanol in a container submerged in an ice bath to precipitate the polymer solution. Finally, the sulfonated precipitated polymer was filtrated and washed with DI water a few times to eliminate the remaining methanol. Finally, under N_2_ environment, it was dried out, first at 80 °C for 12 h and then 150 °C for 6 h [38].

### 2.3. FO membrane Substrates Fabrication via Phase Inversion

Table 1 shows the casting solution compositions with a certain amount of PES and SPES denoted as sample T_1_, T_2,_ and T_3_. PES and SPES were mixed in a sealed glass container with NMP and then stirred for 24 h at ambient temperature. An ultrasonic bath was used for degassing dope polymer solutions for 1 h. Then, the solutions were placed in a desiccator before casting for over 48 h. The casting was conducted using a stainless-steel film applicator (gate height was 200 µm) on a glass plate. Immediately after casting, the substrates were immersed into a water bath for 15 min. The substrates were then stored in DI water and stored in dark, cool conditions (5–8 °C).

### 2.4. Fabrication of Polyamide (PA) Rejection Layer

Preparing a thin film composite membrane was accomplished by forming polyamide active layer on the membrane substrate top surface by IP. First, the substrates were placed in a sealed frame so that the monomer solutions will only be in contact with the top surface. The samples were soaked in 3.4 wt % MPD solution for 2 min, and then the excess MPD solution was removed using filter paper [40]. Next, 0.15 wt % TMC solution in N-hexane was poured onto the substrate top surface for 2 min to form the rejection layer. The resultant TFC-FO membranes were washed with water to eliminate the remaining solution and kept in DI water at 8 °C FO experiments and characterization.

### 2.5. Membrane Characterization

Schottky field emission scanning electron microscope (SEM, Zeiss Supra 55VP, Carl Zeiss AG, Jena, Germany) was used for morphology evaluation. The samples were dried in ambient air for 24 h, then immersed in liquid nitrogen and cut. The samples were coated by Balzers sputter coater (SCD 050, BAL-TEC, Oerlikon Balzers, Bergisch Gladbach, Germany) with a thin film of carbon before SEM imaging. The contact angles of the samples were measured by optical tensiometer (Attension Theta Lite 100, Biolin Scientific, Espoo, Finland). Three to five measurements were recorded for the average value. A digital micrometer (293–330 Mitutoyo, Kawasaki, Japan) was used for sample thicknesses measurement. Porosity (ε) of the membrane samples was measured by determining the net wet (W_1_) and dry mass (W_2_) based on the following equation [41]:

(1) $$\varepsilon =\frac{\left(W_{1}-W_{2}\right)/\rho_{i}}{\left[\frac{W_{1}-W_{2}}{\rho_{i}}\right]\;+\left[W_{2}/\rho_{m}\right]}\;\times 100$$

where $\rho_{i}$ and $\rho_{m}$ are the density of the solvent and membrane, respectively.

Membrane tensile strength was obtained by an LS_1_ tensile testing device (AMETEK, Lloyd Instruments, Ltd., UK). The structural parameter (*S* value) of the membrane was calculated by the following equation by considering membrane tortuosity (τ), porosity (*ε*), and support layer thickness (t):

(2) $$S=\frac{t\tau }{\varepsilon }$$

#### Thin-Film Composite Membrane Characterization

The intrinsic properties of the membrane samples were assessed by RO testing mode. Water permeability (*A* value) was obtained based on the following equation:

(3) $$A=\frac{∆V_{a}}{∆t_{a}\cdot ∆A_{m}\cdot ∆P}$$

The *A* value was calculated by applying hydraulic pressure of 1–5.0 bar onto a feed container with DI water. The amount of permeate water over time is Δ*V_a_*, whereas Δ*P* is the applied pressure variance and Δ*t_a_* and *A_m_* is the affective membrane zone [42].

Membrane sample salt rejection was measured based on the following equation:

(4) $$R=\frac{Cf-Cp}{Cf}\;\times 100\%$$

where *Cf* and *Cp* are the concentration of accumulated salt in the feed and permeate, respectively.

Salt permeability (*B*) of samples was measured by the following equation:

(5) $$\;\;\;\;\;\;\;B=\frac{A\left(1-R\right)\left(\Delta p-\Delta \pi \right)}{R}$$

where *R* is the rejection of the samples and Δ*p* and Δ*π* are the applied hydraulic pressure and the osmotic pressure difference for the membrane samples, respectively.

### 2.6. TFC-FO Performance Tests

FO tests were run on a bench-scale FO unit. The schematic experimental setup is available in a previous study [2]. The setup contained two peristaltic pumps which were connected to the FO testing cell by rubber tubes. The flow rates of both sides were kept consistent at 200 mL/min while each test was run for 20 min. By measuring the average change weight of both the DS and FS tank, water flux (*J_w_*) was determined.

The membranes’ performances were tested under both FO and pressure-retarded osmosis (PRO) modes. The reverse solute flux (RSF) of the membrane during FO performance was obtained by recording the electrical conductivity (EC) when the FS was DI water via a multimeter (CP-500L, Seoul, Korea).

The water flux can be calculated by the following equation in the FO mode [43]:

(6) $$\;J_{w}=\frac{1}{K_{D}}\;\left[ln\frac{A\pi_{D,b}+B}{A\pi_{Fm}+Jw+B}\;\right]\;\mathrm{FO}\;\mathrm{mode}$$

In the PRO mode, the water flux can be calculated by the equation:

(7) $$\;J_{w}=\frac{1}{K_{D}}\;\left[\;\;\;ln\frac{A\pi_{D,m}-Jw+B}{A\pi_{F,b}+B}\;\right]\;\mathrm{PRO}\;\mathrm{mode}$$

*B* is the salt permeability coefficient of the TFC-FO membrane. The *π_Dm_* and *π_Fm_* are the osmotic pressures on the membrane surfaces in the DS and FS containers, respectively*,* whereas *π_D,b_*, and *π_F,b_* are the bulk osmotic pressure of the DS and FS tanks. *K_D_* is the solute resistivity for the diffusion of draw solutes, which can be determined as:

(8) $$K_{D}=\frac{t\tau }{\varepsilon D}=\frac{S}{D}$$

where *τ, t,* and *ε* represent tortuosity, thickness, and porosity of the membrane samples, respectively. *D* is diffusivity or the diffusion coefficient of membranes.

### 2.7. Model Development

**Figure 3:**
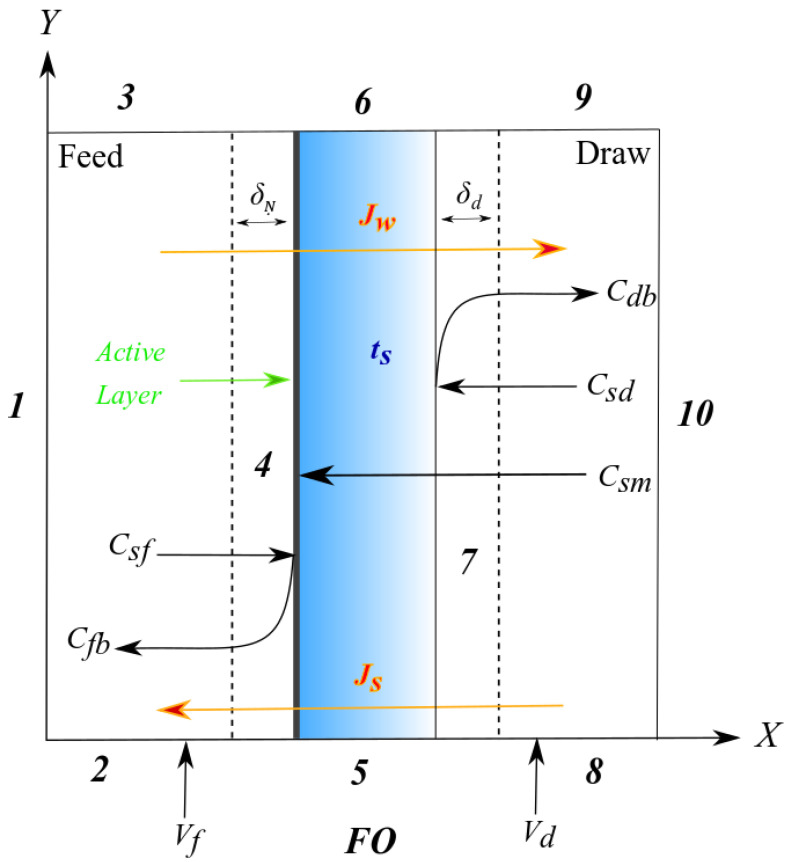
A schematic of the forward osmosis (FO) model diagram. Nomenclature: *J_w_*—water flux, *J_s_*—reverse salt flux, *C*—concentration, *V*—cross flow velocity, *t_s_*—porous thickness, *δ*—concentration polarization thickness; subscripts: *m*—membrane, *d*—draw, *f*—feed, *b*—bulk.

A (computational fluid dynamics) CFD model for the FO was developed via the finite element method by COMSOL Multiphysics^®^ (Version 5.4, COMSOL Inc., Stockholm, Sweden). A schematic of the FO diagram was presented in Figure 3.

#### Governing Equations

To simplify the CFD model, the below assumptions were considered:

- Steady-state;
- Isothermal conditions;
- Flow is incompressible and the laminar flow on the draw and feed solution channels; and
- Thermodynamic equilibrium at the interface of the active layer.

To calculate velocity distribution on the draw and feed solution channels, Navier–Stokes and continuity equations were simultaneously applied [44]:

(9) $$\frac{\partial u}{\partial x}+\frac{\partial v}{\partial y}=0$$

(10) $$\left(u\frac{\partial u}{\partial x}+v\frac{\partial u}{\partial y}\right)=-\frac{1}{\rho }\frac{\partial p}{\partial x}+\frac{\mu }{\rho }\left(\frac{\partial^{2}u}{\partial x^{2}}+\frac{\partial^{2}u}{\partial y^{2}}\right)$$

(11) $$\left(u\frac{\partial v}{\partial x}+v\frac{\partial v}{\partial y}\right)=-\frac{1}{\rho }\frac{\partial p}{\partial x}+\frac{\mu }{\rho }\left(\frac{\partial^{2}v}{\partial x^{2}}+\frac{\partial^{2}v}{\partial y^{2}}\right)$$

*P*, *ρ*, and *μ* are the pressure, solution density, and dynamic viscosity, respectively.

To calculate velocity distribution in the support layer, the Brinkman equation was applied due to high accuracy:

(12) $$-\frac{\partial P}{\partial x}+\frac{\mu }{\varepsilon }\left(\frac{\partial^{2}u}{\partial x^{2}}+\frac{\partial^{2}u}{\partial y^{2}}\right)=u\frac{\mu }{\kappa }$$

(13) $$-\frac{\partial P}{\partial y}+\frac{\mu }{\varepsilon }\left(\frac{\partial^{2}v}{\partial y^{2}}+\frac{\partial^{2}v}{\partial x^{2}}\right)=v\frac{\mu }{\kappa }$$

*ε* and *κ* are the porosity and the pure water permeability of the porous layer, respectively.

To calculate concentration distributions through the draw and feed solution channels, Fick’s equation was applied to both convection and diffusion terms:

(14) $$u\frac{\partial c}{\partial x}+v\frac{\partial c}{\partial y}=D\left(\frac{\partial^{2}c}{\partial x^{2}}+\frac{\partial^{2}c}{\partial y^{2}}\right)$$

where *D* is the diffusion coefficient.

To calculate concentration distributions in the support layer, Fick’s equation was used:

(15) $$u\frac{\partial c}{\partial x}+v\frac{\partial c}{\partial y}=\frac{\varepsilon }{\tau }D\left(\frac{\partial^{2}c}{\partial x^{2}}+\frac{\partial^{2}c}{\partial y^{2}}\right)$$

The boundary conditions were defined for the FO in Table 2 [45].

The mathematical equations have been introduced to predict water and reverse salt fluxes for FO mode based on the bulk concentration of the feed and draw solution, the driving force of the process. It should be noted that the effects of ECP and ICP were considered in these equations to increase prediction accuracy.

Dilutive ICP (FO mode):

(16) $$J_{w}=A\left[\frac{\pi_{db}\exp\left[1-J_{w}\left(\frac{1}{k_{d}}-\frac{S}{D_{d}}\right)\right]-\pi_{fb}\exp(\frac{J_{w}}{k_{f}})}{1+\frac{B}{J_{w}}\left[\exp(\frac{J_{w}}{k_{f}})-J_{w}(\frac{1}{k_{d}}-\frac{S}{D_{d}}\right]}\right]$$

where *A* and *B* are the water and salt permeability coefficient; $\pi_{db}$ and $\pi_{fb}$ are the bulk osmosis pressure; *k_d_* and *k_f_* are mass transfer coefficients through the draw and feed solution channels, respectively; *S* is the membrane structural parameter; and *D_f_* and *D_d_* are diffusion coefficients on the feed and draw solution channels, respectively.

The RSF was evaluated by:

(17) $$J_{s}=B\left[\frac{C_{db}+\frac{J_{s}}{J_{w}}}{\exp(J_{w}K_{r})\exp(\frac{J_{w}}{k_{d}})}-\left(C_{fb}+\frac{J_{s}}{J_{w}}\right)\exp(\frac{J_{w}}{k_{f}})\right]$$

where *C_db_* and *C_fb_* are the bulk concentrations of the feed and draw solution channels.

There are some relationships presented to predict the quantity of mass transfer coefficients. The analytical Leveque solution can predict mass coefficients with high accuracy, based on the following equations:

(18) $$Re=\frac{V\rho d_{h}}{\mu }$$

(19) $$Sc=\frac{\mu }{\rho D}$$

(20) Sh=1.85(ReScdhL)0.33

(21) $$k_{m}=\frac{ShD}{d_{h}}$$

where *Sc* is the Schmidt number, *Sh* is the Sherwood number, *Re* is the Reynolds number, and *d_h_* and *L* are the hydraulic diameter and length, respectively.

## 3. Results and Discussion

### 3.1. Sulfonated Membrane Substrate Characterization

**Figure 4:**
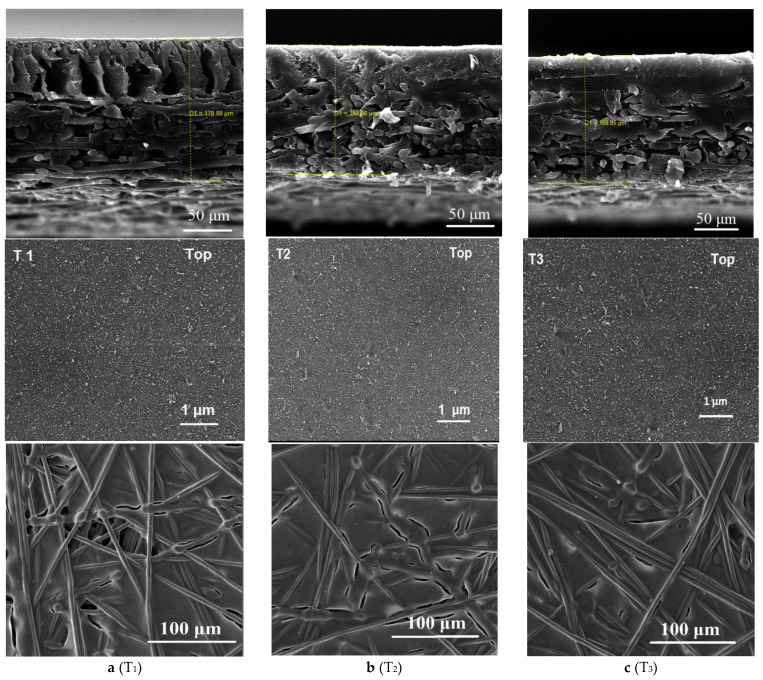
Scanning electron microscopy (SEM) images of cross-section, top surfaces, and bottom surfaces of the fabricated (**a**) PES (no sulfonated polymer), (**b**) SPES (25 wt % sulfonated material) and, (**c**) SPES (50 wt % sulfonated material) membrane substrate samples denoted as T_1_, T_2_, and T_3,_ respectively.

Blending sulfonated materials into pure PES polymer solution was performed to fabricate an enhanced FO TFC membrane with a more hydrophilic substrate. Sulfonation was expected to greatly affect the FO membrane substrate and the performance of the resulting FO TFC membrane. In this study, the substrate morphology and hydrophilicity were first investigated. Figure 4 presents the SEM images of membrane samples fabricated with three different concentrations of sulfonated PES, as described in Table 1.

Figure 4a presents the SEM images of cross-section, top, and bottom surface of the synthesized membrane substrates containing zero SPES content. The occurrence of a large number of macrovoid structures can be seen, which is consistent with an earlier study [23]. However, with the presence of SPES, the macrovoids in the developed sulfonated substrates became noticeably fewer, although they were still indistinctly noticeable at 25 wt %, as revealed in Figure 4b. For the 50 wt % SPES substrate, the SEM images exhibited a fully sponge-like morphology where macrovoids disappeared, as shown in Figure 4c.

The ternary diagram can explain several pathways that could have happened throughout the phase inversion for the polymer solution [46]. Based on the diagram, substrate morphology is a result of the demixing rate in phase inversion. Blending SPES could delay demixing and, as a result, the sponge-like structure would be formed. With an increase in sulfonated material content, macrovoid formation reduced significantly. Meanwhile, in the non-sulfonated sample, instantaneous demixing resulted in forming the macrovoid structure [23,37]. Dope polymer solution viscosity is one of the important factors in membrane fabrication that affects transport characteristics and morphology. Enhancing the polymer concentration increases the solution viscosity. When the solution viscosity increases, a slower mixing is obtained. On the contrary, the mass transport rate during an exchange of solvent and non-solvent decreases, and the polymer chains precipitate slowly. Moreover, the polymer precipitation crosses the binodal curve at higher polymer contents in the ternary phase diagram, leading to the formation of a substrate with a thicker skin layer and lower porosity [45].

Based on SEM images in Figure 4, different degrees of sulfonation did not alter the top and bottom surface of substrates, while the two membranes had noticeable differences in the cross-section morphology. Nevertheless, in the previous study by atomic force microscopy (AFM), it was realized that with the increase in sulfonation concentration, surface roughness decreased [12]. Similar to a previous study, it can be seen that with the increase in sulfonation, traces of finger-like structures in the bottom surface of the sample decreased [23].

Previous studies explored the consequence of sulfonation on substrate thickness [12,47,48]. Several factors can influence the thickness during phase inversion; nevertheless, the thermodynamics of polymer dope solution was found to be the key contributing factor [49,50]. Commonly, casting solution with low thermodynamic instability produces thinner substrate [23]. Based on the SEM images (Figure 4) and membrane substrate thickness measurements in Table 3, higher amounts of SPES blended in the polymer doper resulted in a thinner substrate with a sponge-like porous structure. Despite the lower thickness of the dried membrane substrates, wetting resulted in water absorption and swelling for the sulfonated samples; thus, both the membrane substrates containing SPES (T_2_ and T_3_) were thicker under wet conditions.

Table 3 shows the characteristics of the developed membrane samples. The results show that the porosity and the hydrophilicity of the substrates were enhanced with the increase in sulfonation. The T_1_ substrate contact angle (0 wt % sulfonation) was 65°, while for T_2_ and T_3_, samples were reduced to 45° and 35°, respectively, owing to the improved hydrophilicity. Accordingly, these results indicate that by increasing the sulfonation rate ratio, if applicable, membrane substrates with a higher grade of hydrophilicity can be developed. Additionally, the membrane sample thickness was slightly decreased with an increase in SPES materials. For the neat membrane sample (T_1_) with zero content of SPES materials, the thickness was 178 µm, whereas for the T_2_ and T_3_ samples with 25 wt % and 50 wt % SPES incorporation, substrate thickness was reduced to 163 and 158 µm, respectively.

As FO is not a pressure-based process, the tensile strength of the FO membrane can generally be ignored. However, applying the FO membrane in a module for application at an industrial scale where large water flows are expected could accumulate certain hydraulic pressure during the process. Previous studies confirmed that membrane polymeric film tensile strength can be significantly affected by sulfonation [23,37]. From the results in this study, it was clear that by increasing sulfonation ratios to 50 wt %, the membrane mechanical strength declined substantially. Thus, in this work, to reinforce the overall substrate mechanical strength, the membrane substrate was cast on a nonwoven backing fabric support. The mechanical properties of the membrane samples are presented in Table 3. Increasing the sulfonation decreased the tensile strength and Young’s modulus of the substrates. Due to the use of fabric backing support, the overall mechanical stability of all membrane substrates became reasonable.

**Figure 5:**
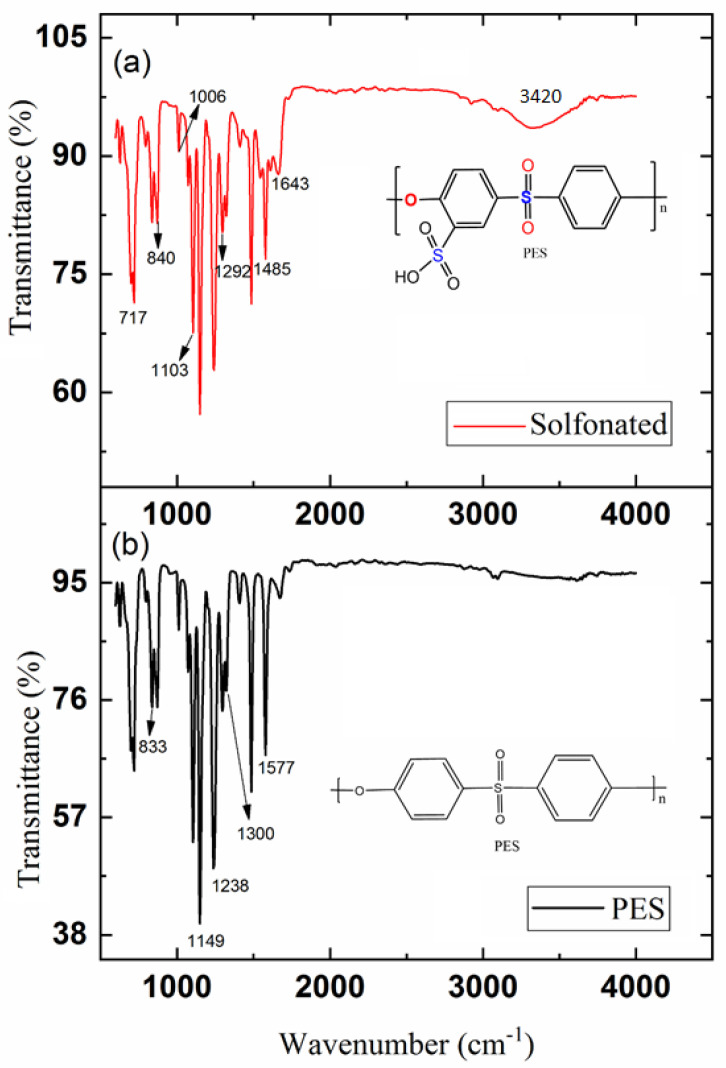
Fourier transform infrared (FTIR) spectra of the membrane substrates for (**a**) SPES (T_3_), and (**b**) PES (T_1_) samples.

Figure 5 shows the comparison of the FTIR spectra of T_1_ (neat sample) and T_3_ samples with 50 wt % of SPES. The spectra contained vibrations of aromatic SO_3_H and sulfonic acid groups appearing at ~1025 cm^−1^ and ~1180 cm^−1^, respectively, confirming the existence of the SO_3_H group on the polymer chains [51,52]. Furthermore, the absorption peak at 3420 cm^−1^ could be attributed to the hydroxyl of sulfonic acid groups, further confirming the existence of sulfonic acid groups in the SPES substrate.

### 3.2. Characterization of TFC FO Membranes

Table 4 presents the structural parameters, transport properties, and rejection performance of the fabricated samples. The water permeability coefficient (*A*) rose when the SPES blending ratio was increased. Different degrees of sulfonation could affect the top surface properties (pore size, interface degree), topologies, and chemical properties, which accordingly affected the IP and development of the rejection layer [12]. Although it is difficult to distinguish the consequence of blended sulfonated materials on IP and rejection layer from comparing the membrane morphologies, water flux performance and salt rejection for the blended and neat membranes can demonstrate the effect of sulfonation on rejection layer properties. For instance, the *A* values for the T_1_ sample (0 wt % SPES) was 1.62 Lm^−2^ h^−1^ bar^−1^ (LMH bar^−1^), and it was raised to 2.21 LMH bar^−1^ and 3.15 LMH bar^−1^ for T_2_ and T_3_, respectively. With an increase in the degree of sulfonation, salt permeability coefficient (*B*) similarly increased. Nevertheless, salt rejection remained at an acceptable level as it was 96.5 % for the T_1_ sample, and for T_2_ and T_3_, the rejections were 94.1%, and 92.8%, respectively. Overall, the rejection of these reinforced membranes on PET fabric were higher compared to the samples in the previous study without backing fabric support. PET fabric as a backing support in this study also may act as a barrier to water flux, but it can be neglected due to the low-thickness and high-porous nature of the PET nonwoven fabric. Furthermore, polymer solution penetrates the fabric during the cast and solidifies during phase inversion. Membrane support polymeric film embedment with the fabric in the bottom surface is more porous (spongy) which may potentially reduce the ICP effect. By comparing these results with previous work, suitable PET nonwoven backing fabric support may break the permeability–selectivity trade-off of the TFC FO membrane and improve its tensile strength without sacrificing the water permeability properties through lowering overall ICP.

In the FO membranes, the *A* and *B* values are intrinsic transport parameters associated with the properties of the active rejection layer. Therefore, a rise in the *A* and *B* parameters with the increase in sulfonation degree is linked to the top skin layer characteristics of the PA rejection layer. As mentioned earlier, an increase in sulfonated material content in the polymer solution leads to a more even skin surface, which could affect interfacial polymerization, leading to the formation of uniform and thinner rejections [37]. Furthermore, a lower degree of sulfonation leads to the formation of rougher top skin surface during phase inversion, which might result in the development of a thicker rejection layer during phase inversion, thus reducing the TFC FO membrane permeability [12].

A lower structural parameter (*S* value) is favorable for FO membranes [53]. The membrane *S* value is associated with the ICP degree and membrane physical properties, such as porosity and thickness. With a rise in the degree of sulfonation, the *S* values of the membrane samples reduced. The *S* value for the T_1_ sample was 10.9 ×10^−4^ m, which reduced to 7.84 ×10^−4^ m and 5.91×10^−4^ m for T_2_ and T_3_, respectively. Sulfonation could therefore result in the formation of a more porous substrate with a lower S value.

### 3.3. TFC FO Membranes Performance

**Figure 6:**
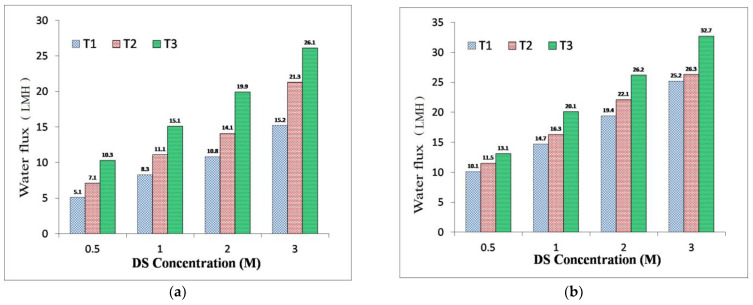
Membrane performance comparison in terms of water flux under (**a**) FO and (**b**) pressure-retarded osmosis (PRO) modes at different NaCl concentrations (DI water as the feed, T_1_ contains 0 wt % SPES blend concentration while T_2_ and T_3_ contain 25 wt % and 50 wt % SPES blend concentrations in the polymer solution, respectively).

Performances for the fabricated membrane samples under FO and PRO modes were evaluated. The DS was 0.5–3.0 M of NaCl and feed solution was DI water. Figure 6a,b show the water flux performance of the TFC FO membrane samples under the FO PRO modes, respectively. As anticipated, the water flux rose with the DS concentration increase for all samples due to a higher DS concentration generating a higher driving force. As shown in Figure 6a,b, with the rise in the amount of sulfonation, the performances of the FO membrane samples were enhanced in both modes, consistently with the membrane *A* values.

Accordingly, in the PRO mode, similar water flux enhancement for the sulfonated TFC FO samples was also observed. For instance, the water flux for the T_1_ membrane (2 M NaCl DS-DI as FS) in the FO mode was 10.8.4 LMH and improved to 19.4 LMH under the PRO mode of operation. However, in the same conditions and molar concentration, the water flux levels under the FO mode for T_2_ and T_3_ were 14.1 and 19.9 LMH, which improved to 22.1 LMH and 26.1, respectively. Under the PRO mode, in this study, water flux performances for T_2_ and T_3_ are only about 30–40% higher than the FO mode of membrane orientation. Generally, in the FO process, the water flux difference between FO and PRO modes is significant by two folds, similar to the T_1_ membrane sample (with no sulfonated polymer) [2,48,49]. However, in this study, the performance difference between FO and PRO is slightly lower for T_2_ and T_3_ samples. This could indicate the insignificant level of dilutive ICP in the FO mode and further confirm the positive effect of sulfonation on the substrates [11,54].

Table 5 also summarizes the membrane performance in terms of water flux, RSF, and specific reverse salt flux (SRSF) in FO and PRO modes of operation. For instance, the T_1_ FO membrane (2 M NaCl as DS) had 10.8 LMH water flux where it was 14.1 LMH for T_2_ and 19.9 LMH for T_3_ under the FO mode. Thus, the results show the membrane flux improvement by sulfonation. A similar enhancement in water flux under the PRO mode was also observed. For instance, the water flux of the T_1_ FO membrane (using 2 M NaCl as DS) was 19.4 LMH, while it was 22.1 LMH for T_2_ and 26.2 LMH for T_3_ under the PRO mode. Based on the membrane performance data presented in Figure 6 and Table 5, it is evident that sulfonation could significantly enhance the membrane water flux performances concerning RSF.

This improvement was most likely due to the changes in the membrane substrate hydrophilicity and porosity. Evaluation of the cross-section morphologies for membrane samples (Figure 4) indicated interesting results. The T_1_ membrane sample with a finger-like pore structure had lower performance in comparison with T_2_ and T_3_ membrane samples, which both possess a sponge-like and denser structure. Based on this finding, the ideal FO membrane morphology can be irrelevant as the FO membranes’ performances can also be enhanced by increasing membrane hydrophilicity. However, in this study, it can be seen that the substrate hydrophilicity enhancement via sulfonation could also alter the substrate morphology into a more sponge-like porous structure.

RSF is an important performance parameter that shows the amount of draw solute diffusing through the membrane reversely [55]. This parameter indicates the permselectivity of the membrane. A high RSF can complicate concentrate management, membrane fouling, and scaling, in addition to the loss of DS [56,57].

**Figure 7:**
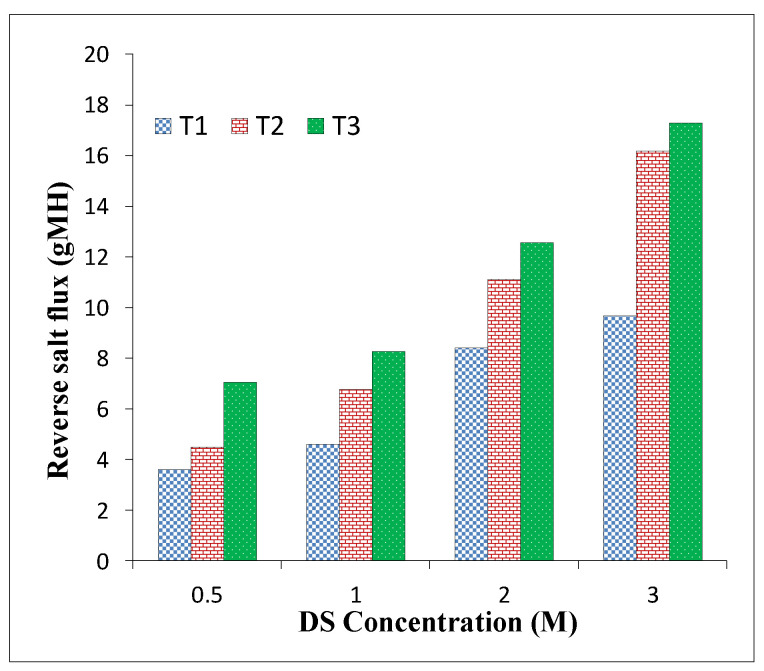
Membranes’ performance in terms of reverse salt flux (RSF) under FO mode with different NaCl molar concentrations as DS and deionized wate(DI) r as FS.

Figure 7 and Table 6 show the RSF of membrane samples in this study under both FO and PRO modes of operation. Even though the water flux increased with the rise in the sulfonation degree, the RSF also consistently increased for all three membrane samples, as expected, due to the trade-off of permeability and selectivity.

The RSF of the T_1_ membrane sample was 8.4 gMH in the FO mode, which rose to 11.1 gMH and 12.55 gMH for T_2_ and T_3_, respectively. Similarly, the RSF for the T_1_ sample was 11.1 gMH, rising to 14.7 gMH and 16.8 gMH for the T_2_ and T_3_ samples with the PRO mode, respectively. Similar to the trend for water flux, RSF was also observed to be higher under the PRO mode compared to the FO mode. To provide more comprehensive information about the combined permeability and selectivity performance of the TFC membranes, the specific RSF (SRSF) can be determined to give a better evaluation of membrane performance under different operating modes. SRSF is defined as the quotient of the RSF and the water flux, which is impartially constant regardless of the DS concentrations [58].

**Figure 8:**
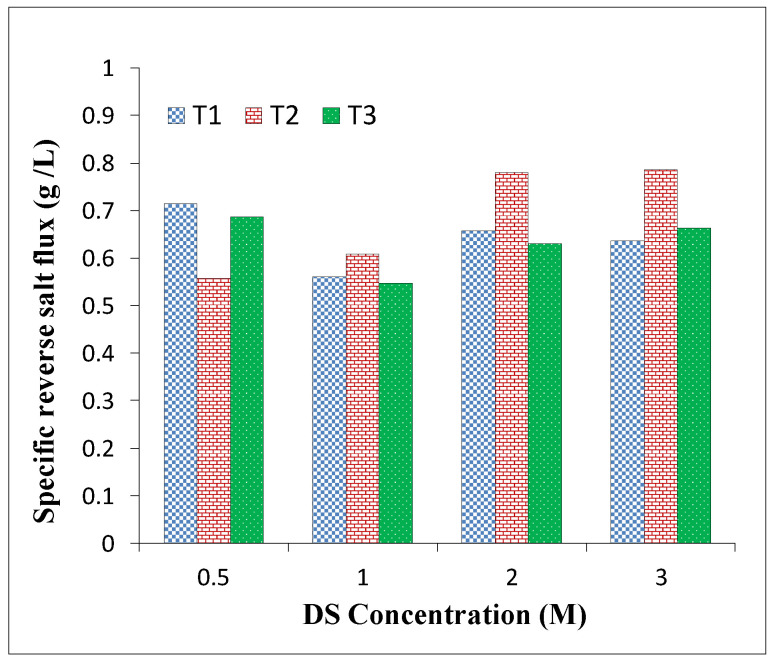
Membranes’ performance in terms of specific reverse salt flux (SRSF) under the FO mode with different NaCl molar concentrations as DS and DI water as FS.

Figure 8 shows the SRSF for the developed membranes in this study. With an increase in sulfonation degree, SRSF was slightly reduced to some extent, but it was constant in the range of 0.5–0.8 g/L.

**Figure 9:**
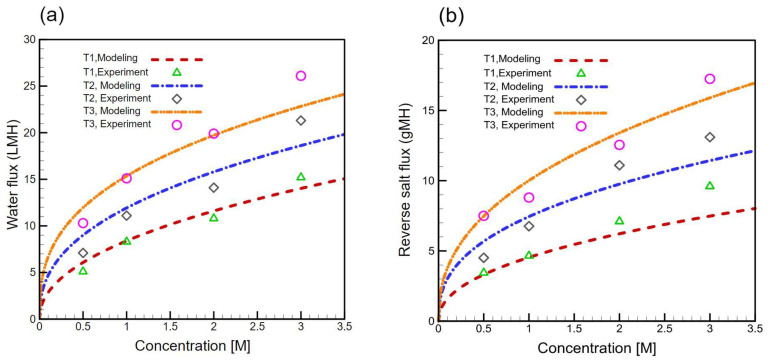
Comparison of computational fluid dynamics (CFD) model and experimental (**a**) water flux (*J_w_*) and (**b**) reverse salt flux (*J_s_*) water flux and RSF results to validate the accuracy of experimental data.

In this study, CFD simulation was performed to see if the sulfonated PES-based TFC membrane behaves in accordance with all phenomena occurring during the FO process. The results of the CFD model and experimental data were compared for all fabricated membranes (T1, T2, and T3) and are presented in Figure 9. Considering all resistivities including ICP and ECP, RSF, and the variable parameters, dynamic viscosity, density, and osmotic pressure, which could vary with different concentrations, our CFD model could calculate the driving force near the actual driving force. In most of the previous studies, the driving force was considered constant along with membrane length, but this is not a correct assumption. In our model, the 2000 concentrations along membrane length were utilized to compute 2000 *J_w_* and *J_s_*; after that, their average was calculated. Additionally, the experimental data resulted from several repetitions. Therefore, the CFD model results have an acceptable agreement with experimental data.

The mathematical equations (Equations (16) and (17)) could not anticipate with high accuracy the amount of *J_w_* and *J_s_* when the concentration is higher than 1 M [62]. A possible reason for the discrepancies is the application of the effect of ECP by calculating the mass transfer coefficients, which could cause errors between CFD and experiment results because the amount of these mass transfer coefficients can be different from the actual quantity of these resistivities. Despite the discrepancies, a good agreement was obtained between the experimental and simulated results.

Table 6 shows the comparison between the results of the current work with previous and commercialized different TFC flat sheet FO membranes. The membranes prepared in this study were only compared with other TFC membranes with woven and nonwoven backing fabric support in literature, to provide a fair comparison. The best-performing membrane prepared in this study was found to be comparable in terms of water flux and SRSF with other TFC membranes in literature. 

## 4. Conclusions

In this study, nonwoven fabric backing-supported TFC FO membranes with PES/SPES substrate were developed. The effect of SPES blending onto the membrane substrate was investigated through changes in morphology, total thickness, hydrophilicity, tensile strength, and TFC membrane performances. Changes in substrate morphology from macrovoid to sponge shape, thinner thickness, and lower membrane hydrophilicity were achieved after SPES blending into the PES polymer material. Furthermore, membrane water permeability increased while the membrane structural parameter decreased, which resulted in better membrane performance under the FO process. The modified TFC FO membrane showed high water flux performance of 26.1 LMH and 32.7 LMH, under FO and PRO modes, respectively, using 3 M NaCl as DS and DI water as FS. This high-performance membrane may be suitable for fertigation applications in the fertilizer-drawn forward osmosis (FDFO) unit; however, developing smarter membranes such as biomimetic substrates may be the answer to elevate the FO process to a commercial scale.

## Figures and Tables

**Table 1:** Compositions of the casting solutions.

Samples	Solution Composition			Sulfonated Polymer Content (%)
	PES (wt %)	NMP (wt %)	SPES (wt %)	
T_1_	15	85	0	0
T_2_	10	85	5	25
T_3_	7.5	85	7.5	50

**Table 2:** Boundary conditions for the FO operation at FO mode.

BC No.	NS–Feed	CD–Feed	Brinkman—Porous	CD–Porous	NS–Draw	CD–Draw
1	$u_{f}=0$	$\partial c_{f}/\partial x=0\;$	-	-	-	-
2	$v_{f}=v_{f0}$	$c_{f}=c_{f0}$	-	-	-	-
3	$\partial u_{f}/\partial y=0\;$	$\partial c_{f}/\partial y=0\;$	-	-	-	-
4	$u_{f}=J_{w}$	$J_{s}$	$u_{p}=J_{w}$	$J_{s}$	-	-
5	-	-	$u_{p}=0$	$\partial c_{p}/\partial y=0\;$	-	-
6	-	-	$u_{p}=0$	$\partial c_{p}/\partial y=0\;$	-	-
7	-	-	$\partial u_{f}/\partial x=0\;$	$c_{p}=c_{d}$	$u_{d}=J_{w}$	$c_{p}=c_{d}$
8	-	-	-	-	$v_{d}=v_{d0}$	$c_{d}=c_{d0}$
9	-	-	-	-	$\partial u_{d}/\partial y=0\;$	$\partial c_{d}/\partial y=0\;$
10	-	-	-	-	$u_{d}=0$	$\partial c_{d}/\partial x=0\;$

BC = boundary condition; CD = convection and diffusion equations; NS = Navier–Stokes equations.

**Table 3:** Characterization of the membrane substrates with different degrees of sulfonation.

Membrane ID	Thickness (µm)	Porosity (%)	Contact Angle (°)	Mechanical Properties (with Backing Fabric)		
				Tensile Strength (MPa)	Modulus (MPa)	Elongation at Break (%)
T_1_	178 ± 2.0	71 ± 2	65 ± 1	42.1	115.2	39.2
T_2_	163 ± 3.0	77 ± 3	45 ± 1	36.1	82.2	36.2
T_3_	158 ± 2.0	82 ± 2	35 ± 2	33.2	55.6	43.3

**Table 4:** Membrane samples transport properties and structural parameters.

Sample ID	^a^ Water permeability (*A*)		^b^ Salt Permeability *B* (10^−7^ m/s)	NaCl Rejection (%)	^c^ S (×10^−4^ m)
	LMH bar^−1^	×10^−12^ m/s Pa			
T1	1.62 ± 0.15	4.5 ± 0.5	1.6 ± 0.1	96.5	10.9
T2	2.21 ±0.1	6.13 ± 0.5	3.59 ± 0.1	94.1	7.84
T3	3.15 ±0.15	8.75 ± 0.5	6.25 ± 0.15	92.8	5.91

^a^ Assessed in the RO setup (applied hydraulic pressure of 10 bar and DI water as FS). ^b^ Assessed in the RO setup (applied hydraulic pressure of 10 bar and 200 ppm NaCl as FS). ^c^ Assessed in the FO setup using 1 M NaCl as the DS with DI water as FS.

**Table 5:** Thin-film composite (TFC) FO membrane performance under FO and PRO modes using 2 M NaCl as DS and DI as FS modes.

Membrane ID	FO Mode			PRO Mode		
	Water Flux (LMH)	RSF (gMH)	SRSF (g/L)	Water Flux (LMH)	RSF (gMH)	SRSF (g/L)
T_1_	10.8	8.4	0.65	19.4	11.1	0.57
T_2_	14.1	11.1	0.78	22.1	14.7	0.66
T_3_	19.9	12.55	0.63	26.2	16.8	0.64

**Table 6:** Comparison performances of flat sheet TFC FO membranes on different fabric support.

Membrane Types/Materials/Support Fabric			Water Flux (LMH)		Specific Reverse Salt Flux (g/L)		DS NaCl (M)	FS	References
			FO Mode	PRO Mode	FO Mode	PRO Mode			
TFC flat-sheet (HTI)	Psf	Polyester mesh	16.8	33.1	0.44	0.55	1.0	DI	[59]
TFC flat-sheet (HTI)	Psf	Polyester mesh	13.0	N/A	0.81	N/A	2.0	DI	[14]
TFC flat-sheet membrane	CE ^1^	N/A	37.6	N/A	0.17	N/A	1.0	DI	[60]
TFC flat-sheet membrane	Psf	N/A	12.0	20.5	0.40	0.31	1.0	10 mM NaCl	[13]
TFC flat-sheet membrane	Psf	PET nonwoven	15.1	N/A	-	-	1.0	DI	[14]
* TFC PAO ^2^	PES	Compacted woven mesh	16.1	-	0.43	-	0.5	DI	[8]
TFC FO	PES	Permeate spacer fabric	17.1	21.0	0.47	0.46	2.0	DI	[61]
TFC FO	PES	Nonwoven PET fabric	19.9	26.2	0.63	0.64	2.0	DI	Present work

^1^ Cellulose ester. ^2^ Pressure-assisted osmosis. N/A: without or not available. * Note: For the TFC-PAO membrane, applied hydraulic pressure was 5 bar.

## Data Availability

Data are available on request from the corresponding author.
